# Dangerous Liaisons: Gammaherpesvirus Subversion of the Immunoglobulin Repertoire

**DOI:** 10.3390/v12080788

**Published:** 2020-07-23

**Authors:** Monika A. Zelazowska, Kevin McBride, Laurie T. Krug

**Affiliations:** 1 Department of Epigenetics and Molecular Carcinogenesis, Science Park, The University of Texas MD Anderson Cancer Center, Smithville, TX 78957 USA; mazelazowska@mdanderson.org (M.A.Z.); kmcbride@mdanderson.org (K.M.); 2 HIV and AIDS Malignancy Branch, Center for Cancer Research, National Cancer Institute, National Institutes of Health, Bethesda, MD 20892, USA

**Keywords:** gammaherpesvirus, Epstein–Barr virus, EBV, Kaposi sarcoma herpesvirus, KSHV, HHV-8, human herpesvirus 8, murine gammaherpesvirus, MHV68, latency, B cells, immunoglobulin repertoire, geminal center, receptor editing, somatic hypermutation, isotype class switching, clonal expansion

## Abstract

A common biologic property of the gammaherpesviruses Epstein–Barr Virus and Kaposi sarcoma herpesvirus is their use of B lymphocytes as a reservoir of latency in healthy individuals that can undergo oncogenic transformation later in life. Gammaherpesviruses (GHVs) employ an impressive arsenal of proteins and non-coding RNAs to reprogram lymphocytes for proliferative expansion. Within lymphoid tissues, the germinal center (GC) reaction is a hub of B cell proliferation and death. The goal of a GC is to generate and then select for a pool of immunoglobulin (Ig) genes that will provide a protective humoral adaptive immune response. B cells infected with GHVs are detected in GCs and bear the hallmark signatures of the mutagenic processes of somatic hypermutation and isotype class switching of the Ig genes. However, data also supports extrafollicular B cells as a reservoir engaged by GHVs. Next-generation sequencing technologies provide unprecedented detail of the Ig sequence that informs the natural history of infection at the single cell level. Here, we review recent reports from human and murine GHV systems that identify striking differences in the immunoglobulin repertoire of infected B cells compared to their uninfected counterparts. Implications for virus biology, GHV-associated cancers, and host immune dysfunction will be discussed.

## 1. Introduction

Epstein–Barr virus (EBV) and Kaposi sarcoma herpesvirus (KSHV) are two of the seven oncogenic viruses associated with human cancers. These viruses target and subvert many key processes intrinsic to the B cell in addition to modulating cytokines and interactions with other cells in the microenvironment. The germinal center (GC) of lymphoid tissues is a hypermutagenic environment that poses a formidable barrier to the virus due to the high rate of B cell turnover. Decades after EBV and KSHV were first identified, we still do not understand the role of the GC in long-term infection and oncogenesis. It is important to understand the impact of infection on the mutation of the immunoglobulin (Ig) gene and the selective processes that the infected cell undergoes to gain access to long-lived memory B cells. The Ig gene can serve a molecular barcode to inform the origin of infection, the degree of engagement and selection within the GC, clonal expansion, and disease progression. The model gammaherpesvirus MHV68 enables spatial and temporal resolution of B cell engagement during primary infection of mice. Here, we review the intersection of gammaherpesviruses (GHV) with B cells and highlight recent discoveries using high-throughput and single-cell sequencing technologies that indicate the GHVs do not obey the standard rules of Ig diversification and selection.

## 2. B Lymphocytes: A Major Reservoir of Gammaherpesvirus Latency and Lymphoproliferations

Herpesviruses co-evolved with the animal kingdom and, in doing so, typically colonize and persist for the lifetime of their host without undue disease burden. The complex and dynamic strategy of chronic infection used by these enveloped, double-stranded DNA viruses involves latency, a quiescent infection that does not produce infectious particles, accompanied by intermittent periods of productive lytic replication. Viruses of the GHV subfamily have been identified in many mammals, spanning wild and livestock ruminants [1], rodents [2], cats, bats, primates [3] and humans.

A key biological property of GHVs is their ability to engage the lymphocytes they infect to undergo a proliferative program of expansion and molecular reprogramming while bypassing host checkpoints. Their propensity to cause cancer is an unfortunate outcome of this strategy of persistence in the host. Etiological associations of GHVs with lymphoproliferative disorders, in addition to cancers of epithelial and endothelial cell origin are noted within natural hosts and upon cross-species transmission in primates [4,5] and ruminants. This review will focus on the B cells targeted by the oncogenic GHVs of humans, Epstein–Barr virus (EBV, HHV-4) and Kaposi sarcoma herpesvirus (KSHV, HHV-8), in addition to the small animal model pathogen, the B lymphotropic murine gammaherpesvirus 68 (MHV68, γHV68, MuHV-4) of murid rodents.

### 2.1. Epstein–Barr Virus, Prototype Member of the Lymphocryptovirus Subfamily of the Gammaherpesviruses

Denis Burkitt reported in 1963 [6] that children in equatorial Africa suffered from a high rate of lymphoma that was later associated with malaria endemic areas [7]. Anthony Epstein acquired these tumors and was successful in cultivating single-cell suspensions with the assistance of Yvonne Barr, leading to the first documentation of herpesvirus particles in these tissues by electron microscopy in 1964 [8]. This enveloped, double-stranded DNA virus named Epstein–Barr Virus (EBV) was the first discovery of a human tumor virus. Werner and Gertrude Henle were additional pioneers who confirmed the association of EBV with Burkitt Lymphoma and later made the etiological links to infectious mononucleosis and nasopharyngeal carcinoma [9]. 

Over the last fifty years, the remarkable ability of EBV to immortalize primary B lymphocytes in cell culture has identified viral proteins and non-coding RNAs that coordinate an orchestrated cellular reprogramming to drive rapid proliferation, termed latency III. Gene expression analysis of infected B cells from healthy individuals and tumor samples reveal multiple latency programs (latency 0, I, II, III) that enable EBV access to long-term cellular reservoir but also places target cells at risk for further genetic insults.

One of the most intriguing and worrisome aspects of EBV is its high worldwide (95%) prevalence in the adult population. Once acquired via saliva in the oral cavity, this virus is never cleared; and the individual becomes a life-long carrier. Most children and a large percentage of adolescents undergo an asymptomatic infection. Infectious mononucleosis (IM), an extended period of debilitating malaise and swollen lymph nodes, accompanies primary EBV infection in 25–50% of adolescents. The virus readily infects B cells in the Waldeyer’s ring of the nasopharyngeal tonsil tissue either via direct infection or after limited replication in the mucosal epithelium. Upon initial infection, EBV is found in multiple B cell subsets of primary lymphoid tissues, but after the resolution of the proliferative phase of infection, the virus resides in a long-lived differentiated B cell subtype, the isotype class-switched memory B cell, for the life of the individual [10]. Memory B cells likely provide the virus an opportunity for homeostatic maintenance and episodic reactivation from latency for transmission between hosts. The role of the germinal center in this process will be discussed below in Section 3.

EBV is associated with numerous types of cancers of B cells: Burkitt lymphoma (BL), Hodgkin lymphoma (HL), and diffuse large B cell lymphoma (DLBCL). Notably, the incidence of EBV-associated cancers is markedly increased in populations with immune dysfunction. EBV drives B- lymphoproliferative disease in solid or stem cell transplant recipients (post-transplant lymphoproliferative disease, PTLD) and in people living with HIV, particularly those with severe CD4^+^ T cell lymphopenia. EBV co-infection is also seen in primary effusion lymphoma (PEL), which is an aggressive B cell lymphoma caused by KSHV and strongly associated with HIV infection. BL, HL, DLBCL and PEL are dramatically higher in people living with HIV (PLWH), even in the post-ART era. EBV has causal links to lymphoproliferations of other blood lineage cells including Natural Killer cells and T cells, in addition to epithelial cells of gastric carcinoma and nasopharyngeal carcinoma. Features of EBV-associated lymphomas have been recently reviewed in Shannon-Lowe, et al. [11]. The degree of association with EBV and the latency gene expression program differs with each lymphoma, but a striking commonality of BL, HL, and DLBCL is that they have features of being GC experienced. 

### 2.2. Kaposi Sarcoma Herpesvirus, Prototype Member of the Rhadinovirus Subfamily of the Gammaherpesviruses

Kaposi sarcoma (KS) was first identified as a rare vascular neoplasm of elderly men by the physician Moritz Kaposi in 1872 and was later recognized as an aggressive variant in children of central and southern Africa. With the development of the HIV epidemic, KS became a major AIDS-defining illness [12]. The causative agent, Kaposi sarcoma herpesvirus (at that time named Kaposi sarcoma-*associated* herpesvirus, KSHV), was discovered in the laboratory of Yuan Chang and Patrick Moore using a technology of subtractive hybridization that identified pieces of a herpesvirus DNA sequence in diseased, but not normal skin tissues [13]. Ethel Cesarman joined the effort to characterize this new GHV and identified KSHV in eight lymphomas of HIV+ patients [14]. KSHV is genetically and biologically distinct from EBV. KSHV does not immortalize primary B cells and lacks many of the proteins and non-coding RNAs encoded by EBV. However, KSHV employs its own homologs of many cellular proteins that drive oncogenic processes and transformation. In the 25 years since the landmark discovery of this second oncogenic GHV, KSHV has taught the field new and distinct lessons from EBV about oncogenic processes in B cells and the role of inflammation in neoplasia [15,16].

KSHV and its associated cancers is the leading cause of morbidity and mortality in persons living with HIV (PLWH) world-wide. Yet there is little understanding of primary KSHV infection of the host, much less the first encounter with B cells, due to that lack of a defining syndrome when the virus is acquired. In contrast to the high prevalence of EBV, KSHV prevalence in adults is under 10% in the US and Europe, ~30% in the Mediterranean, while reaching 90% in parts of sub-Saharan Africa [16]. Transmission between adults in non-endemic areas may involve sexual transmission, but oral transmission via saliva is also possible [17]. In areas where KSHV is endemic, KSHV shedding in the saliva is frequent and ~30% children become seropositive by five years of age, strongly supporting a saliva-borne horizontal mode of transmission [17]. KSHV is also detected in the tonsils and adenoids in children and adolescents [18]. Mucocutaneous KS is common in all geographic locations and in both endemic and epidemic KS suggesting oral transmission of KSHV is of primary importance [19]. The tonsillar and adenoid tissues of the oral cavity are a likely cite of initial infection and lytic amplification during KSHV pathogenesis. KSHV infects multiple cell types including B lymphocytes, monocytes, dendritic cells, endothelial and epithelial cells. The interplay of different cell types that serve as reservoirs of infection within the host is not well understood. 

KSHV infection of PLWH drives four independent, and sometimes concomitant disease manifestations: Kaposi sarcoma (KS), an endothelial cell-derived neoplasia of the skin and viscera; primary effusion lymphoma (PEL); a subset of multicentric Castleman disease (MCD); and KS inflammatory cytokine syndrome (KICS) [20]. PEL and MCD are two types of B cell lymphoproliferative diseases. KSHV+ MCD B cells have characteristics of plasmablasts that localize to the mantle zone of the lymph nodes. These nonmalignant polyclonal cells express IgM, lambda light chains and have not undergone somatic hypermutation [21,22]. In contrast, PEL is a monoclonal B cell lymphoma frequently detected in body cavities of peritoneal, pericardial or pleural spaces. PEL generally expresses the plasma marker syndecan 1 (CD138) and lacks most B cell markers [23]. While surface Ig expression is typically absent, somatic hypermutation is present providing evidence the cell traversed the GC, [24,25]. PEL is notable for the frequent occurrence of co-infection with KSHV and EBV. 

### 2.3. Murine Gammaherpesvirus 68 Infection of Mice, an Animal Pathogen System 

MHV68 is the best characterized small animal model of GHV infection and pathogenesis. As a member of the rhadinovirus arm of the GHV subfamily, MHV68 is genetically closer to KSHV, but shares with EBV and KSHV the properties of driving primary B cell proliferation and lymphoma development in the host [26]. MHV68 is the prototype strain of MuHV-4 and was isolated from bank voles in a survey of pathogens from Slovakia [2]. MHV68 and related strains have been isolated from murid rodents across Europe and readily infects laboratory mice with a similar pathogenic process [27]. Transmission in wild rodents is likely via saliva. In addition, a sexual route of transmission is supported by epidemiological studies and transmission between co-housed lab mice, with evidence of shedding and primary infection of the genitalia [28]. 

MHV68 undergoes a short period of replication at the mucosal site of primary infection prior to the establishment of latency in multiple subsets including macrophage, dendritic cells, and lymphocytes. Macrophage or dendritic cells are vehicles of dissemination to the draining lymph nodes where B cells are infected [29]. Hematogenous dissemination to secondary lymphoid tissues such as the spleen tissues requires B cells [30] and leads to a rapid expansion of monocytes and lymphocytes. MHV68 infects multiple B cell subsets, expanding most rapidly in the follicular GC compartment [31,32,33,34,35,36]. At the peak of latency, ~14–18 days post-infection (dpi), the virus is most frequently in germinal center B cells. By six weeks of infection and for the life of the host, isotype class-switched IgD- B cells are the major reservoir of latency [32,33], as found for EBV. Host and viral factors that influence the access of MHV68 to the germinal center compartment have been recently reviewed by Johnson and Tarakanova [37].

As with a healthy human population infected with the human GHVs, the vast majority of immune competent mice do not develop MHV68 cancers. However, prolonged infection in the context of immunosuppression may manifest as lymphohyperplasia. Lymphomas that are clonal and Ig light chain restricted develop in aged mice or upon treatment with cyclosporin A [26]. Infected mice that lack CD8 T cells develop lymphohyperplasia and these cells express the plasma cell marker CD138 [38]. B220+ B cell lymphomas that are MHV68+ develop in the lungs of IFNγ receptor-/- mice 5–12 months after infection [39]. In addition, MHV68 can immortalize fetal liver B cells ex vivo, and the latency-associated proteins LANA and v-cyclin, which are conserved with KSHV, are required [40]. Detailed analysis of the Ig repertoire is lacking for all MHV68-associated lymphoproliferation and tumor models.

## 3. Germinal Center Processes That Shape B Cell Evolution and Gammaherpesvirus Latency

### 3.1. Dynamic Molecular Events as the B Cell Traverses Compartments of the Germinal Center

#### 3.1.1. Overview

B cells are a critical part of the adaptive immune system. They express unique immunoglobulin (Ig) receptors that are individually evolved through three distinct processes, V(D)J recombination, somatic hypermutation (SHM), and class switch recombination (CSR). The negative selection processes of tolerance remove B cells producing self-reactive Igs, while positive selection during the GC center process of affinity maturation results in the evolution and expansion of B cells that express Igs with antigen specificity. During development and selection Igs are expressed, by alternative splicing, as either secreted antibody or a membrane-bound form as part of the B cell receptor complex (BCR). Sensitive to antigen cross-linking, the BCR signals activation and survival signaling pathways into the nucleus. It also binds and mediates antigen uptake and processing for presentation to helper T cells. BCR specificity therefore plays a role in competing for T cell help. With these functions, the BCR plays a critical role in determining which B cells survive, proliferate and differentiate into various subsets. Therefore, in B lymphocytes, the immune receptor sequence rather than development stage is key to differentiation and outcome. This is reflected by certain B cell subsets (B-1, etc.) having recurrent or restricted BCR properties. Within the GC, the BCR–antigen interaction is a key determinant to drive clonal expansion and evolution. Because the BCR acts as a physiologic genetic lineage barcode, tracing the BCR repertoire can inform the origin and fate of the infected cell. Since the BCR plays a substantial role in B cell pathologies from neoplasia to autoimmunity, the connection of GHVs to certain repertoire could provide mechanistic insight into viral infection routes and related B cell pathologies.

#### 3.1.2. Immunoglobulin Assembly and Somatic Hypermutation

The Ig variable region is initially assembled during B cell development in the bone marrow through RAG1/2- recombination of heavy chain (Igh) variable (V), diversity (D) and joining (J) gene segments on the heavy chain (IGH), and (V) to (J) gene joining of the light chain [41,42]. Antigen interaction is predominantly through complementarity-determining region (CDR) loops with three CDRs on both heavy and light chains. CDR1 and CDR2 are encoded entirely within (V) segments while CDR3 is created by the V(D)J junction of the heavy chain. Successful assembly of the BCR results in tonic signaling that triggers B cell transit to the periphery. During immune responses, Ig repertoire can evolve improved affinity through affinity maturation in the germinal center (GC) reaction. Activated B cells migrate into germinal center structures where they proliferate and undergo SHM. This process introduces random, nontemplate nucleotide mutations into the Ig variable gene [43]. Mutations can increase BCR affinity and give cells an advantage for antigen interaction and uptake. This facilitates T follicular helper (Tfh) cell interaction and signals, which are required for survival and clonal expansion [44,45]. Antigen selection plays a key aspect during the GC reaction. B cells expressing BCRs with higher antigen affinity are able to remain in the GC reaction and outcompete other B cells for antigen [46]. As cells continue through the GC reaction, they can differentiate into antibody secreting plasma cells and memory B cells [47].

#### 3.1.3. Isotype Class Switching

CSR is a deletional recombination reaction that replaces one heavy chain constant region exon for another. This results in antibody isotype switching without altering variable regions and results in an antibody that retains antigen specificity but has altered antibody effector function. Although SHM and CSR are different mechanisms, they are initiated in the nucleus by activation-induced cytidine deaminase (AID) a mutator enzyme that introduces uracil lesions into transcribed single-stranded (ss) DNA at the Ig loci [48,49]. Subsequent mutagenic repair results in either a mutation during SHM or DNA double-strand break (DSB) during CSR. Successful recombination and DSB repair by the non-homologous end-joining pathways completes the CSR process, resulting in isotype-switched antibodies. 

#### 3.1.4. Genome Instability

DSBs are among the most dangerous lesions to genome stability since they are potential substrates for major chromosome translocations and genomic rearrangements. AID and RAG1/2 activity directly induce DSB that if mis-targeted or aberrantly repaired can result in genome instability. Most mature B lymphomas carry translocations with the Igh locus being a common partner (MYC, BCL6, etc.) [50]. A common example is the IGH/MYC translocation which is the etiology of Burkitt lymphoma. In this case, the strong IGH enhancer deregulates expression of the MYC oncogene. These translocations bare hallmarks of either aberrant RAG1/2 mediated V(D)J or AID mediated CSR events as translocations junctions occur at J exons or IGH switch regions [51,52]. In the case of IGH/MYC, AID itself causes the DSB breaks at both MYC and the IGH locus and so is directly responsible producing the DSB translocation substrates [53,54]. Although AID activity is normally focused at the Ig genes, it is present at a wide range of transcribed non-Ig loci [55] and potentially can cause genome wide DSBs. Break analysis performed on a genome wide scale found AID-dependent DSBs on a variety of transcribed genes including those associated with recurrent translocations in lymphomas [56,57]. AID expression and activity are strictly regulated. Multiple transcriptional and post-translational mechanisms modulate AID activity or targeting [58]. AID mis-expression induces genome instability and cancer [59]. Within B cells, deregulation of AID protein levels [60,61] or post-translational modifications that control activity [62,63] result in genome instability and chromosome translocations. Therefore, viral factors that induce AID and RAG1/2 or disrupt the regulatory mechanism of these proteins could produce oncogenic lesions that contribute to associated lymphomas.

#### 3.1.5. Receptor Editing

During B cell development, cells that have self-reactivity are removed during the process of tolerance. This can occur during early bone marrow development (central tolerance) or in the periphery after events such as SHM (peripheral tolerance). BCR signaling functions to regulate the tolerance process which can alter specificity by receptor editing or eliminate the cell via anergy or cell death. Receptor editing is the predominant central tolerance mechanism but may also be activated in the periphery. This process can salvage the B cell by altering the light chain sequence of the BCR. RAG1/2 and the recombination machinery reactivate and reassemble the light chain, resulting in a different specificity that may not recognize self-antigen. Unlike the IGH, the light chain can be encoded by two different genes, Kappa (IGκ) or Lambda (IGλ). However, the assembly process is orderly and controlled by epigenetic factors. The Kappa gene assembles first and only proceeds to Lambda if Kappa cannot be successfully assembled or self-reactivity persists [64]. In wild-type mice, the vast majority of B cells in the mature B cell compartment express a Kappa chain [65]. In humans, the ratio of Kappa to Lambda is usually closer to equal [66].

### 3.2. B Cell Differentiation and Selection Processes in the Context of GHV Infection

#### 3.2.1. Epstein–Barr Virus

**Figure 1:**
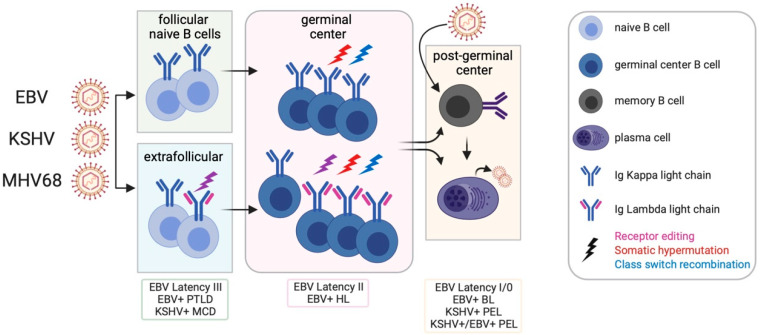
EBV, KSHV, and MHV68 are B cell-tropic members of the GHV that subvert the B cell differentiation processes in which they participate. GHVs infect naïve and extrafollicular B cells. Infected B cells enter the germinal center with a BCR repertoire that is distinct from the uninfected GC B cells. Infected B cells undergo somatic hypermutation and class-switch recombination, while undergoing proliferative expansion and likely evading typical selective pressures. In addition, B cells infected with KSHV and MHV68 undergo receptor editing. GHVs exit the GC as an isotype class-switched B cell or a PC. Direct infection of post-GC memory B cells is a potential route to the class-switched memory compartment. Plasma cell differentiation drives GHV reactivation. Created with BioRender.com.

Our knowledge of primary EBV infection comes from the analysis of B cell subsets in the tonsillar lymphoid tissue, lymph nodes and peripheral blood of infected individuals in combination with following the process of EBV immortalization of naïve B cells in culture. David Thorley-Lawson proposed the germinal center model wherein EBV-infected cells participate in the germinal center compartment to gain access to the memory B cell compartment and maintain proper homeostasis in the host [10]. In this model (Figure 1), distinct latency programs facilitate the entry into the GC (latency III growth program), the transition across the GC (latency II default program) and exit of EBV-infected cells from the GC as memory B cells (latency I/O). EBV-associated diseases and lymphomas reflect particular latency programs and suggest that transformation events arise from distinct stages of the engagement of EBV with differentiating B cells: latency III for PTLD and a subset of diffuse large B cell lymphomas; latency II for HL; and latency I/0 for BL.

The latency III program of EBV that drives the initial lymphoproliferative phase produces nuclear antigens (EBNAs 1, 2, 3A, 3B, 3C, LP), surface proteins (LMPs 1, 2), and numerous non-coding RNAs including miRNAs and circular RNAs. The function and activities of these viral factors have been reviewed in depth elsewhere [67,68,69,70,71,72,73]. Global transcriptional and metabolic profiles in primary B cells upon new infection with EBV in cell culture paint a picture of latency III whereby the virus ramps up and then drives any type of B cell through a rapid phase of proliferation and pushes a B cell to a state of differentiation with plasmablastic features [74,75,76]. The viral effectors of the latency III program coordinate the proliferation and survival of the newly infected B cell by providing surrogate receptor signaling and modulating genes that regulate B cell differentiation, including MYC, PU.1, EBF1, and IRF4 [75], in addition to promoting latent viral gene expression [77]. 

EBV infection upregulates AID expression and, as detailed in Section 4 below, the LCL clones bear evidence of somatic hypermutation in the immunoglobulin locus (Table 1). A select few cells acquire the properties of immortalization to ‘grow out’ as lymphoblastoid cell lines (LCLs). These cells are CD27+, IgD+ non-isotype class switched and express IRF4, AID, PRDM1, XBP1, and PAX5, but not Bcl-6 [75,78,79]. LCLs serve as a model for the oncogenic potential of EBV that manifests in the absence of T cell pressure, as too often occurs for transplant recipients with PTLD and other lymphomas in PLWH.

Next, via the latency II program, EBV usurps the process of B cell differentiation to gain access to the long-lived memory B cell that is maintained for much of the host’s lifespan. The process of memory B cell differentiation takes place in the germinal center within the follicles of lymphoid tissues. In latency II, LMP1 and LMP2A function as ligand-independent receptors to provide surrogate CD40 and BCR survival signals, respectively; in combination with EBNA1, EBERs, and BART miRNAs. EBV-infected B cells are physically located in the GC of tonsillar tissue and bear the surface markers CD10, CD77, CD38, CXCR4, CXCR5. They express bcl-6 and AID [90] and undergo a process of proliferation and death, consistent with the GC reaction [91]. In a transgenic mouse model that limits LMP1 and LMP2A expression to the germinal center, the depletion of NK and T cells leads to plasmablast expansion, and upregulation of inflammatory cytokines, and mortality [92]. EBV-infected cells of HL express a latency II program. Interestingly, the Hodgkin/Reed–Sternberg cells of HL typically lack BCR expression due to non-productive rearrangements [93]. 

In latency I, only EBNA-1 is expressed to maintain the non-integrated viral genome as an episome. In latency 0, when the infected IgD- CD27+ memory B cells are not undergoing cell division, viral proteins are not detected. EBV+ BL express only EBNA1. These cells exhibit a germinal center (GC) centroblast gene signature that express IgM, CD10 and Bcl-6, yet with a rearranged and somatically mutated Ig sequence suggesting aberrant exit from the germinal center.

With regard to actions that directly impact the immunoglobulin locus, several viral proteins influence AID expression levels. EBNA3C of latency III directly upregulates the expression of the Ig mutator activation-induced cytidine deaminase (AID) and slightly elevates IgH somatic hypermutation of primary B cells in culture [94]. LMP-1 upregulates AID in EBV-negative B cell lines [95] yet EBNA2 induction in a LCL led to a decrease in AID expression [96]. In addition, the coincidence of Burkitt lymphoma with malaria suggests that coinfection with the causative agent *Plasmodium falciparum* promotes EBV oncogenesis. Interestingly, *P. falciparum* infection drives immune activation to increase the GC B cell population and within GC B cells, AID is upregulated and increases c-myc translocations [97,98,99,100].

#### 3.2.2. Kaposi Sarcoma Herpesvirus

KSHV infects and drives the proliferative expansion of primary tonsillar B cells [86,87,101,102]. Since KSHV infection does not drive immortalization of primary B cells, these studies are limited in duration. However, observations in primary B cells in culture are useful to model early stage infection events in B cells of the host, and they may relate to lymphoproliferations observed for MCD. Upon tonsillar B cell infection ex vivo, KSHV is found in the IgM+ λ light chain-expressing B cells. MCD B cells which harbor KSHV are IgM+ with a λ light chain receptor and lack evidence of somatic hypermutation [21,22]. This is consistent with an extrafollicular origin that might be exacerbated due to immune dysfunction in PLWH [103].

A detailed temporal analysis by Totonchy et al. [87] revealed that the virus initially infects B cells expressing either Igκ and Igλ light chains, but drives a rapid switch to Igλ light chain usage over a 10 day time course. Receptor editing is a highly regulated process following sequential rearrangement attempts at each light chain loci to generate a B cell with a functional and properly reactive BCR [64]. This suggests that KSHV might edit the BCR in the peripheral lymphoid tissues and lead to some potential autoreactive or non-functional B cells outside of the bone marrow. As detailed and discussed below, Igκ light chain bias was also recently reported by us [88] and Collins et al. [89] for MHV68-infected B cells from mice at the peak of latency in the spleen. It is unclear by what mechanism KSHV drives receptor editing and for what purpose this might serve the virus in the host. KSHV infection upregulates RAG1/2 recombinase expression [87], but the viral factors specific to this dysregulation are unknown. In addition, multiple molecular events are required to make the chromatin accessible and recruit the recombination machinery to drive Ig gene rearrangement [104,105]. Thus, receptor editing might reflect a much larger reprogramming event in response to KSHV infection. 

PEL is a rare subtype of non-Hodgkin lymphoma (NHL) with a plasma cell gene expression profile [106]. Unlike the polyclonal MCD lymphoproliferation, PEL is a clonal lymphoma [107,108] and is often dually infected with EBV and KSHV. In a comparative study by Chadburn et al. [23], MCD and PEL both express B cell master regulators BLIMP1 and IRF4 along with the Ki67 proliferation marker but not PAX5 or Bcl-6. MCD retains IgM and variable expression of CD30 and CD27 while PEL lacks many typical B cell surface receptors including CD19, CD29, CD22 and surface Ig, but express CD30 and CD138. Taken together, there appears to be a block in terminal plasma cell differentiation for both lymphoproliferative disorders, with MCD being of extrafollicular origin in contrast to a germinal center/post-germinal center origin for PEL. KSHV may infect multiple B cell subsets within and outside of the follicle in lymphoid tissues of healthy individuals.

While KSHV infection is required for a diagnosis of PEL, at least 80% of PEL is co-infected with EBV that expresses EBNA-1 and EBV miRNAs in addition to the KSHV latency genes. Recent studies suggest that KSHV and EBV cooperate with regard to lymphomagenesis. Co-infection of humanized mice with KSHV and EBV led to lymphoproliferations in splenic follicles [109]. Positive cell lines derived from these mice shared features with PEL including the upregulation of BLIMP1 and IRF4 and downregulation of CD20. KSHV was also associated with an increase in EBV lytic gene expression. In cell culture studies, EBV enhanced KSHV infection of peripheral B cells and the dually infected cells that exhibited higher levels of KSHV latent gene expression prevailed in transformation assays [110]. EBV enhances KSHV viral load and viability of PEL cells via EBNA-1 [111,112].

The KSHV latency program has been defined in lymphoma cell lines and artificial latency systems by comparing the profile of uninduced to drug-induced reactivation conditions [113,114]. Latency-associated genes are constitutively expressed while lytic genes are responsive to reactivation stimuli. Similar patterns are observed by in situ hybridization analysis of MCD and PEL [115]. The majority of cells are latent with KSHV gene expression limited to LANA, vFLIP and v-cyclin, Kaposin, and 25 miRNAs, with the addition of v-IRF-3 detection in MCD and PEL. The functions of each viral protein have been extensively reviewed elsewhere [15,115,116,117]. In brief, LANA is the functional homologue of EBV EBNA-1 that maintains the viral episome and it subverts tumor suppressors and cooperates with H-ras. B cell-specific expression of LANA leads to germinal center expansion and proliferation of IgM+IgD+ B cells [118]. KSHV vFLIP blocks Fas-mediated death and activates NF-κB to promote survival. The viral-cyclin complexes with CDK6 and impairs the cdk inhibitor p27/Kip1 [119]. Kaposin B stabilizes transcripts for cytokines such as IL-6 that is a hallmark of KSHV-pathologies [120]. 

In a latent cell culture system or in a KSHV neoplasia, there exists a subpopulation of KSHV-infected cells that either undergo full lytic reactivation or express a low level of genes ascribed to the lytic cycle such as K1 and vIL6 [113]. The contribution of this lytic, active subpopulation to lymphomagenesis is unclear, but potentially contributes to immune evasion, paracrine factors and viral load. KSHV K1 sequesters the BCR and K15 blocks BCR signaling [121], while vIL-6 promotes survival of PEL cells [122]. KSHV encodes additional proteins and non-coding RNAs that function to prevent innate immune detection and antiviral responses. For instance, K3 and K5 downregulate surface proteins such as MHC-I that enable immune evasion [123,124]. Thus, KSHV directly alters the biology of the B cell it infects, while also shaping the microenvironment. 

With regard to direct effects on the Ig locus, expression of the potent NF-κB activator vFLIP upregulates AID while KSHV miRNAs, K12-11 and K12-5 contribute to post-transcriptional suppression of AID, perhaps to counter AID-mediated suppression of KSHV reactivation [125]. Other points of intersection with the germinal center have been deduced from in vivo mouse models. Transgenic mice expressing the KSHV latency locus under the viral endogenous promoter exhibit multiple phenotypes including increased B cell activation, marginal zone expansion, and increased CD138+ plasmablasts [126]. KSHV miR-K12-11 is an ortholog of host miR-155 that was found to promote B cell expansion and complement for loss of miR-155 in vivo [127,128,129]. Single-cell analysis will better define how viral gene expression profiles alter B cell differentiation and germinal center programming in cell culture and patient samples.

#### 3.2.3. Animal Models to Investigate the Interplay of GHVs with B Cell Biology

Animal models of gammaherpesvirus pathogenesis are needed due to the strict tropism of EBV and KSHV. The phenotypes of transgenic mice expressing proteins from EBV and KSHV are quite informative regarding their oncogenic properties and their singular impacts on primary B cells in a host microenvironment [130,131]. For instance, latency proteins such as LMP1 and LMP2A of EBV, the vFLIP and the entire latency locus of KSHV, in addition to the lytic protein kinase ORF36 have been found to subvert normal B cell differentiation when expressed in a dysregulated manner outside of the context of infection [126,132,133,134,135,136,137]. However, caution is warranted when making inferences regarding the roles of these factors in germinal center participation and B cell reprogramming in the context of whole viral infection. Many viral proteins have opposing actions. Constitutive expression by a strong signaling protein can lead to aberrant B cell behavior and does not reflect the dynamic waves of signaling that link to cell interactions and physical movement through the follicle and within the light and dark zones of the germinal center [46]. In addition, the CD4 and CD8 T cell response that controls cells expressing viral antigens is absent in these models.

Primate GHV pathogenesis in the context of simian immunodeficiency virus (SIV) is the closest model for human GHV-associated diseases. Rhesus rhadinovirus and rhesus lymphocryptovirus from naturally infected rhesus macaques are detected in lymphomas that arise in the context of SIV or SHIV infection [138]. In addition, experimental infection of primates with primate GHVs leads to the colonization of cells, immune response, and pathologies that parallel their human GHV counterparts [3,139,140]. Primate pathogens systems are important to refine knowledge of virus–host interactions at the organismal level and they can provide important pre-clinical evaluation of novel interventions [141]. Cost and ethical considerations, in addition to limitations such as genetically tractable animals, are a barrier to widespread adoption of pathogenesis studies in primates.

MHV68 is a well-developed small animal pathogen system to query whole virus behavior in the context of a primary infection, with the added physiologically relevant wrinkle that the virus is facing the host immune response to itself. *Mus musculus* is predominately used for laboratory studies. The virus has been isolated in numerous wild murid rodent populations, with a similar course of pathogenesis noted between inbred mice and bank voles in laboratory settings [27]. MHV68 encodes ~80 homologs of lytic and latent factors found in the KSHV genome [142,143]. As with all herpesviruses, it encodes unique proteins and non-coding RNAs that likely perform analogous functions achieved by different means in other GHVs. For instance, the M2 latency gene of MHV68 induces the expression of cytokines IL-6 and IL-10 by the host [144], while KSHV encodes a vIL-6 and EBV encodes a vIL-10 that each promote proliferative expansion and skew the immune response. Thus, biology common between the mouse and human GHV can illuminate conserved evolutionary strategies that are likely further refined by the human GHV. Approaches to test how factors encoded by the human GHV influence the infected B cells include gain of function experiments that introduce human GHV genes into MHV68 or genetically complement MHV68 mutants [145,146,147,148,149]. The analysis of EBV and KSHV in humanized mice is an exciting advancement that enables some degree of B cell interactions with cognate T cells [141,150,151]. 

Several recombinant viruses have been engineered to track MHV68 in vivo. Real-time replication can be imaged in the context of a live, whole animal that is infected with a recombinant MHV68 expressing luciferase reporter under the control of the lytic M3 promoter [152,153]. The cells that MHV68 has passed through en route to a different cell reservoir can be traced using a recombinant virus that switches from mCherry to eGFP expression upon the infection of transgenic mice expressing Cre recombinase from a cell type-specific promoter [36,154,155]. A recombinant virus engineered to express a fusion protein of histone H2B with YFP generates a strong nuclear signal for identification of lytic and latent cells by fluorescence microscopy and flow cytometry [156]. The direct fusion of β-lactamase to the C-terminus of the viral protein mLANA enables MHV68-infected cells that express LANA to be detected upon exposure to a fluorogenic substrate [34].

Multiple B cell subsets spanning naïve, transitional, marginal zone (MZ), follicular, germinal center (GC), isotype class-switched memory, and plasma cells harbor MHV68 [31,32,33,34,35,36]. The largest B cell subsets that harbor MHV68 at the peak of splenic latency are GC B cells followed by MZ B cells [31]. MHV68 is detected in MZ B cells prior to entering the white pulp of the follicles. Disruption of MZ B cells with the sphingosine-1-phosphate receptor agonist FTY720 reduced virus entry into the white pulp, suggesting the virus infects this subset prior to GC B cells [31]. 

MHV68 is detected in the GC of follicles by in situ detection of viral miRNAs that are constitutively expressed [157] or by immunofluorescent detection of a reporter gene under the control of the strong CMV IE promoter [158]. GC involvement at the peak of MHV68 latency has been confirmed by numerous orthogonal investigations. At 14–18 dpi, the virus is detected most frequently in PNA+ CD19+ B cells with the GC surface markers GL7 and CD95. At late times post-infection, the virus is found in cells with memory phenotypes, IgD- B cells class switched to cell surface IgG, IgA, or IgE [32] or CD19+ IgD- CD38^hi^ [34]. CD40+ B cells are more frequently infected with MHV68 than their CD40- counterparts at late stages of infection in CD40+/− mixed bone marrow chimeric mice [33]. This body of data demonstrates that MHV68-infected B cells participate in the GC at the peak of splenic latency and then accumulate in isotype class-switched B cells consistent with memory phenotypes. This preference for class-switched memory B cells as a reservoir of long-term latency is a strong biologic parallel with EBV.

As with KSHV and EBV, stimuli that drive B cell activation such as cross-linking of surface Ig cue MHV68 reactivation from latency [159,160,161]. Terminal differentiation of a memory B cell that harbors a GHV to a plasma cell is a physiologic cue for reactivation. Impaired plasma cell differentiation upon loss of specific host [162] or viral factors [163] leads to a reduction in MHV68 reactivation. One common molecular feature of GHV reactivation is the responsiveness of the regulatory regions of the viral ‘lytic switch’ transactivators to the spliced form of X-box binding protein (XBP-1s), a host factor that supports Ig production [161,164,165]. Interestingly, the master regulator IRF-4 but not XBP-1 was found essential for MHV68 reactivation in vivo [166].

MHV68 latency is significantly disrupted in the absence of host factors that are critical mediators of B cell differentiation including NF-κB [167], STAT3 [168], DNMT1 [169], and the plasma cell regulators IRF4 [166] and PRDM1 [162]. The T follicular helper (Tfh)-IL-21 receptor axis is also critical for MHV68 latency in the spleen [170,171], demonstrating a requirement for cognate B–T cell interactions in the GC. B cell-specific loss of the host factor IRF-1 reduces MHV68 latency and GC expansion and reduces the activation of the tyrosine phosphatase SHP-1, another host determinant of latency [172,173].

Infection of mice with MHV68 has been noted for the induction of abnormal antibody responses [174,175]. Virus-specific antibodies do not peak until 3–4 weeks pi. Polyclonal antibodies not relevant to MHV68 may be autoreactive to the host or non-specific, of unknown reactivity. The induction of these seemingly irrelevant antibodies coincides with the peak of splenic latency and are impacted by virus-driven processes. For example, the incidence of autoimmune thrombocytopenia is dependent on MHV68 latency establishment [176] and host-reactive antibodies are decreased when the viral protein kinase is not active [177]. Host factors that promote MHV68 colonization of the germinal center also correlate with an increase in self-reactive antibodies [172,173].

The genetic tractability of MHV68 permits a robust functional analysis of viral factors in acute replication, latency and reactivation in specific cell types. The latency program of MHV68 was defined by qRT-PCR studies of bulk tissue and isolated B cell subsets from infected mice [31,178]. The functions of key viral factors in pathogenesis have been reviewed in detail elsewhere [142,143]. Here, we will highlight functions of viral factors that are critical to B cell latency. 

mLANA is a functional homolog of KSHV LANA that promotes episomal maintenance of the genome in vivo [148,149]. mLANA also impairs p53-induction of cell death during lytic infection [179,180]. MHV68 LANA targets NF-κB via E3 ubiquitin ligase activity for degradation. Loss of this function enables mLANA to support latency but blocks expansion in the germinal center compartment [181]. The viral M2 has route-dependent roles in latency establishment and reactivation, and drives IL-10 production and plasma cell differentiation upon expression in primary B cells [144,163,182]. The viral cyclin (v-cyclin) of MHV68 promotes reactivation from latency by a molecular override of the tumor suppressor, cyclin-dependent kinase inhibitor p18(INK4c) [183,184]. The viral bcl-2 functions to block apoptosis and autophagy [185] and loss of bcl-2 impairs latency in both immature and transitional B cells and reactivation from latency in the spleen [35]. In addition to viral proteins, the non-coding RNA TMER4 of MHV68 promotes dissemination from the lymph node to the spleen to promote latency establishment in the IgM-CD38+ memory compartment [186]. Genetic complementation by the EBER1 non-coding RNA of EBV is a striking example of functional conservation [147].

The immunophenotype of MHV68-driven lymphoproliferations is not well defined. Lymphomas that develop in aged or cyclosporin-treated mice are clonal and Ig light chain restricted [26]. The S11 latent cell line established from cyclosporin-treated mice was IgM+ MHCII+ with a low level of B220 [187]. Lymphohyperplasia that develops in CD8 T cell-deficient mice expresses the plasma cell marker CD138 [38]. MHV68 latent cell lines derived from fetal liver immortalization have a preplasmablast profile, B220+ CD19+ IgG2a+ IgD-/IgM- Igκ+ CD5- c-kit- CD43- and cause tumors when transferred into immune deficient mice [40]. In addition, mLANA and v-cyclin are necessary for MHV68 immortalization of primary fetal liver B cells [40]. Taken together, as seen with the human GHV, viral-dependent gene expression drives lymphoproliferation in the context of immune deficiency and these lymphoproliferations have markers indicative of aberrant B cell differentiation.

## 4. Immunoglobulin Bias in Gammaherpesvirus Infection

### 4.1. Epstein–Barr Virus

EBV infects both naïve and memory B cells in culture irrespective of the Ig isotype [188]. In vitro studies demonstrated that EBV can drive newly infected naïve B cells into the memory B cell phenotype [79]. This ability does not rely on the antigen selection process during the GC reaction, since the in vitro system is lacking components of the GC environment. When PBMCs are sorted into naïve (IgD+ CD27-), switched memory (IgD- CD27+) and non-switched memory (IgD+ CD27+) B cells and subsequently infected with EBV to generate LCL, mutations accumulate in Ig variable regions of naïve cells (Table 1). Additionally, expansion of certain clones dominates the repertoire of infected cells. 

The effect of EBV infection on the B cell repertoire was studied in infectious mononucleosis (IM) patients, where 1 in 2 memory B cells might be infected [189]. Single-cell analysis followed by BCR sequencing of circulating EBV+ memory B cells (CD27+) from peripheral blood show diverse utilization of IGHV gene segments and IGHV gene families. Studies in vivo rely on a low number of analyzed sequences [80,81,82] (Table 1). The infection status of individual B cells was based on RT-PCR detection of the viral EBER1 transcript. The mutational profile of EBV+ cells indicates that they are GC experienced. One feature of the antigen selection process is the accumulation of replacement mutations in antigen-interacting CDR regions of Igs and a higher R/S (replacement to silent) ratio in CDRs than the structural framework regions of V gene segments. EBV-infected memory B cells accumulate mutations in Ig genes in a similar rate to non-infected cells [82], with R/S ratios higher in CDRs [80,81,82]. A seemingly contradictory report indicating that EBV+ B cells do not persist in the GC structure was based solely on localization markers in a few patients [190]. An additional study that provided strong evidence for the GC origin of infected memory cells showed exclusion from non-switched memory B cells (CD27+ IgD+ IgM+) based on the rarity of detection. EBV resides preferentially in switched memory cells in the periphery [81]. Taken together, both the mutational profile and the isotype of the BCR in EBV-infected B cells indicate that they originate in the GC reaction and undergo a selection process similar to uninfected cells. 

While bias towards a particular BCR repertoire in EBV+ circulating memory B cells has not been uncovered, high levels of autoimmune-related Ig accumulate in sera of IM patients [83,84]. IGHV4-34-encoded Ig detected by anti-Id antibody 9G4 [191] is found at high level in patients with EBV and systemic lupus erythematosus (SLE). Further, CLL patients dually infected with EBV and CMV, present exclusive utilization of IGHV4-34 in circulating B cells [192]. Ig encoding IGHV4-34 are self-reactive against a conserved carbohydrate on the surface of red blood cells and other cell types [193]. 

Due to the autoimmune properties of the human IGHV4-34 gene segment, B cells expressing them are excluded from GC and memory B cell compartments and the levels are low in sera of healthy individuals [194,195]. Even though they constitute up to 10% of naïve B cell repertoire of tonsils, IGHV4-34 expressing cells do not differentiate to plasma cells, likely due to tolerance mechanisms [196]. This tight restriction is disrupted in SLE, PLWH, IM and many other lymphoproliferative diseases [84,197,198] where self-reactive antibodies are detected in switched (IgG) plasma cells. 

An association between Burkitt lymphoma (regardless of EBV status) and a biased BCR repertoire was confirmed in patients’ samples and cell lines derived from BL patients [199,200,201] (Table 2). Moreover, clear evidence indicates preferential usage of certain IGHVs in EBV-infected cases [202]. Apparent bias in IGHV gene utilization is detected in EBV+ endemic BL (eBL), where IGHV4-34, IGHV3-23 and IGHV1-69 is overrepresented when compared to the repertoire of normal B cells. However, earlier studies on smaller number of patients do not observe this bias [203]. In eBL, mutations are more frequent in the Ig CDRs than framework regions (when compared to EBV- sporadic BL), providing evidence that they experience an ongoing antigen selection process [201]. Similarly, in EBV+ BL16 and ELI-BL cell lines derived from BL patients, SHM occurs constitutively [204,205]. Apart from eBL, elevated serum levels of IGHV4-34 are detected in EBV-associated nasopharyngeal carcinoma [84].

Human IGHV4-34 is a well-characterized V gene segment and is known to produce autoreactive antibodies. One of the intrinsic features of its germline sequence is the presence of an N-linked glycosylation motif N-X-S/T (N-asparagine, X-any amino acid but proline, S- serine, T–threonine) in the CDR2 of the Ig variable domain. In general, N-glycans play an important role in immunity especially when harbored by the Fc region of Ig. In this case, glycosylation in the variable domain changes the antigen specificity [207]. In eBL, accumulation of glycosylation in the Ig-variable domain was reported. It results from preferential usage of IGHV4-34 and, most importantly, from the appearance of novel sites introduced by somatic mutations [203]. Acquisition of glycan modification in the antigen binding region of the BCR may lead to inappropriate activation and proliferation of B cells in the GC. That may secure survival of B cells with pathological reactivity.

### 4.2. Kaposi Sarcoma Herpesvirus

In vivo studies of the BCR repertoire of KSHV-infected B cells focus on KSHV-associated pathologies (Table 2). In multicentric Castleman disease (MCD), KSHV is restricted to IgM λ light chain-expressing plasmablasts that do not carry somatic mutations in Ig genes. Infected cells derived from naïve cells exhibit a high degree of polyclonality and reside in the mantle zone of B cell follicles [21,22]. In B lymphocyte cell lines derived from PEL, utilization of λ light chain prevails. As a consequence, all λ-expressing cells carry non-productive rearrangement of κ light chain. Studies identify IGHV genes that are expressed in PEL samples from AIDS patients and PEL cells lines derived from these patients. Sequence analysis showed SHM with a high R/S ratio in CDRs indicating a selection process [24,25]. Thus, PEL is likely of a post-GC B cell origin with a gene expression profile similar to malignant plasma B cells [106].

Studies showed that KSHV is able to infect tonsillar B cells [86,208], activated peripheral blood B cells [209], and cell lines. In vitro infection of human tonsillar cells with KSHV indicates that virus persists in Igλ+ B cells [86]. In a time course analysis, the virus was found to infect Igκ+ lymphocytes and transition through an intermediate state with both Igκ+Igλ+, to become predominantly Igλ+ [87]. KSHV infection is accompanied by induction of RAG1/RAG2 expression enabling further rearrangements of Ig loci and expression of Igλ. KSHV+ cells have biased lambda V gene usage with higher utilization of IGLV4 family (Table 1). In healthy donors, this family is expressed at very low frequency [194,210,211,212]. An increased rate of non-productive rearrangements of IgL transcripts in Igλ-expressing infected cells indicates ongoing rearrangement events on the lambda locus [87].

### 4.3. Murine Gammaherpesvirus 68

MHV68 infects B cells independent of BCR specificity in vitro [36,143,213,214]. However, in vivo Ig sequencing studies of infected B cells suggest that BCR specificity does influence infection dynamics. In addition, MHV68 is present in B cells with an Ig repertoire distinct from uninfected cells.

The first study to suggest that BCR repertoire influenced infection dynamics in vivo used mice that were able to generate a mixed population of wild-type and transgenic B cells [213]. The authors used a transgenic SW_HEL_ mouse system [215], where 10–20% of splenocytes express a HEL (hen-egg lysozyme) specific BCR that is capable of SHM and CSR. They found that MHV68 was excluded from the HEL specific B cells [213]. Although MHV68 was able to infect HEL+ CD19+ B cells in vitro, the virus was excluded from these cells in vivo despite the presence of MHV68 in other GC cells. The exclusion from HEL specific cells occurred even under HEL immunization conditions that induced robust GC expansion of the transgenic HEL GC B cells [213]. This led to the intriguing conclusion that MHV68 is not directly infecting GC cells in a random manner.

Two recent studies used a traceable MHV68 infection mouse model to analyze the Ig repertoire of infected cells [88,89]. A transgenic virus which expresses histone H2B fused to EYFP fluorescent protein (MHV68-H2BYFP) [158] was used to infect mice and isolate MHV68+ B cells. At day 17–18 post-infection, the stage of maximum latency expansion in the GC, the studies compared Ig repertoire from the infected and non-infected cells. This was done on both the single cell level as well as NGS sequencing of a large GC population. Significantly, the studies reported a repertoire bias on both the light and heavy chains with similar characteristics in the infected populations (Table 1). While non-infected mice or the MHV68 negative cells from the infected mice predominantly expressed the Igκ light chain (>90%), nearly 50% of MHV68-positive cells expressed Igλ, indicating that the cells had undergone receptor editing. 

In addition to light chain bias, there was also a significant divergence of mouse IGHV gene usage. Ighv1-82 was the most prominently used IGHV by the MHV68 negative population, likely reflecting the ongoing immune response. Significantly, in the MHV68+ population, Ighv10-1 was highly utilized (~20% of all MHV68+ B cells), with little representation of Ighv1-82 in the MHV68+ population. The bias for Ighv10-1 was not only observed recurrently within individual mice, but also spanned three individual mouse facilities [88,89]. Furthermore, Collins et al. [89] examined MHV68+ plasma cells, observing the biased Ig repertoire and prominent Ighv10-1 utilization. Mouse Ighv10-1 together with closely related Ighv10-3 are sole members of Ighv10 family in the C57Bl/6 strain. Other mouse strains including BALB/c and lupus-prone MRL/lpr express different alleles that belong to that family. Expression of Ighv10 family members is maintained at a low level in naïve, unchallenged and healthy mice [216].

Zelazowska et al. [88] examined clonal expansion and overlap in detail, finding very different dynamics between the MHV68+ and MHV68- populations of individual mice. MHV68+ cells were much more confined in the use of IGHV genes. Significant clonal expansion was apparent in the infected population. Very few clones represented a substantial portion of the MHV68+ population. In contrast, the MHV68- population was much more diverse without dominance by any clone. Remarkably, there was very little overlap of clones between the MHV68- and MHV68+ populations even when 20,000 cells were sequenced by NGS. This indicated that the MHV68+ population in the GC was not derived from the infection of existing GC cells. The confined repertoire and recurrent use of Ighv10-1 suggests that some aspect of the BCR is favored and selected for during expansion. While in vitro studies suggest BCR sequence is ancillary to viral entry, it may have an important role in vivo. 

The confined repertoire also suggests that the virus is using a particular subset of B cells to traffic to the GC. The GC is an open structure and any cell that traffics through the follicular zone can make contact and potentially join the GC. MZ cells in particular have been implicated in trafficking MHV68 to the GCs as mice deficient in MZ cells are unable to substantially colonize the GC [31,36]. Subsets of MZ cells are capable of undergoing the GC reaction [217,218]. The GC reaction is normally driven by BCR antigen affinity and Tfh signals. The SHM in MHV68+ cells did not display a physiologically relevant difference between MHV68+ and MHV68- populations [88,89]. Mutation location was significantly biased towards the antigen-binding CDR loops, an indication of antigen selection, since mutations are randomly generated, but clones with improved affinity were antigen selected [88]. Furthermore, the CDR3 length and charge characteristics as well as isotype switching were all similar between the MHV68+ and MHV68- populations. Altogether, MHV68+ cells undergo affinity maturation and CSR in the GC. However, the stark difference in clonal populations and remarkable bias of Ighv10-1 usage in MHV68+ cells suggests that the virus subverts the normal GC selection processes. In general, Ig bearing Ighv10 gene segments are overrepresented in the anti-nucleic acid antibody pool and are capable to bind its antigen regardless of CDR-H3 sequence [219]. Autoantibodies that confer their binding via IGH10 gene segments were previously described [220,221,222,223]. 

The MHV68+ cell Ig sequence analysis of clonal groups demonstrated that SHM was ongoing during GC expansion. Bias of mutation frequency in the CDR regions provides evidence of antigen selection. Analysis of antibodies coded from MHV68+ cells revealed a lower incidence of self-reactivity and anti-viral specificity compared to the MHV68- population [88,89]. The antigen specificity of the MHV68+ B cells remains to be determined. The repertoire analysis studies suggest that rather than a passive passenger, MHV68 is an active participant in the evolution and selection of the BCR in the GC. The implications of that repertoire on viral pathogenesis and reactivation remain to be determined.

## 5. Conclusions and Future Directions in the Field

### 5.1. GHV GC Model

The Thorley-Lawson GC model for EBV beautifully integrated known processes of B cell differentiation with the immunophenotypes and viral gene expression profiles of infected cells in healthy and IM patients [10]. The coordinated and staged expression of EBV’s unique arsenal of viral proteins and non-coding RNAs align with a sophisticated strategy evolved to engage the germinal center to gain access to a resting non-proliferating memory compartment. EBV is the prototypic lymphocryptovirus, but has a distinct biology and pathology from the prototypic rhadinovirus, KSHV. MHV68 is largely colinear with KSHV and is a genetically tractable model pathogen that enables sophisticated tracking of infected B cells as they navigate changes in the immune repertoire in response to their own intrusion of the host. Here, we have reported on several new studies that reveal extreme bias in the Ig repertoire of B cells upon rhadinovirus infection. This has led us to refine the existing EBV GC model to incorporate a potential role for extrafollicular events preceding and perhaps independent of the GC, in addition to the recent findings of non-standard behaviors of MHV68 in the GC [88,89].

In Figure 1, we propose a model whereby GHVs engage multiple B cell subsets including naïve and extrafollicular B cells. GHV-infected cells undergo proliferative expansion, SHM and CSR in the GC. B cells infected with KSHV and MHV68 also undergo receptor editing. For MHV68, we propose that the virus accesses the GC by inducing survival and proliferation in infected GC B cells that would normally be excluded from the GC. GHV-infected cells exit the GC as an isotype class-switched B cell or a PC. Direct infection of memory B cells is also a possibility. The key details that support this expanded model for the GHV are as follows:

1. GHVs infect follicular and extrafollicular B cells [31,36]. The MZ is an established extrafollicular site that may provide a source of IgM+ memory B cell and plasmablasts observed in GHV-associated diseases [21,22,224].
2. The Ig repertoire of MHV68-infected cells is distinct from uninfected cells, indicating that infection of B cells in GC is not a stochastic event [88,89]. There is very little clonal overlap between the uninfected and infected cells [88]. This suggests that the virus enters the GC via a B cell with a distinct repertoire, and that entrant then expands in the GC.
3. The extreme and recurrent clonal expansion of Ighv10-1 indicates either a predilection of infection or a product of MHV68 infection that has a selective advantage in the GC [88,89].
4. B cells infected with KSHV and MHV68 exhibit a bias towards Ig lambda light chain expression. The high depth analysis of Zelazowska et al. [88] captured clones in the process of Igκ to Igλ switching, and is supported by observations of a skew in lambda light chains of GC B cells by Collins et al. [89]. This complements observations of dual Igκ+ Igλ+ tonsillar cells upon new KSHV infection with concomitant induction of the expression of RAG1/2 recombinase [87]. We propose that rhadinoviruses drive receptor editing in the periphery.
5. The Ig of MHV68-infected cells demonstrates SHM [88], consistent with SHM observed for GHV+ PEL and EBV+ B cells in the periphery.
6. The BCR of the infected GC cells is not biased toward viral antigens or reactivity with self [88,89], but whether truly non-specific remains to be determined.
7. MHV68+ B cells undergo isotype class switching in the GC [88].
8. MHV68 ultimately resides in isotype class-switched GC-derived memory B cells [32], as found for EBV in peripheral blood [10].
9. The differentiation of a memory B cell to PC leads to infectious particle production. BCR cross-linking of surface Ig mimics a memory B cell that encounters its cognate antigen to cue terminal differentiation to a plasma cell. Cross-linking the BCR drives the reactivation of EBV, KSHV, and MHV68 in cell culture [159,160,161,164]. Loss of factors that drive plasma cell differentiation reduces MHV68 reactivation from the splenic reservoir in vivo [162,163,166].

This GHV GC model requires further experimental validation in all GHV systems. Unbiased NGS approaches are now available to link surface phenotypes and gene expression profiles with the recombination and mutation profile of the Ig locus, the ultimate molecular barcode.

### 5.2. Implications of Ig Repertoire Bias

The Ig genes that encode the BCR of a B cell clone can be studied to determine what selection, recombination, mutagenic, and differentiation processes a B cell has undergone. Each GHV examined to date demonstrates a bias in the Ig repertoire of the cells they infect. These exciting observations indicate active GHV subversion of the processes that drive and regulate Ig rearrangement and mutation. Here, we discuss new concepts to consider moving forward: (Section 5.2.1) Does the virus skew the Ig repertoire of the B cell reservoir to benefit the long-term latency of the virus? (Section 5.2.2) What is the role of Ig receptor bias in autoimmunity and responses to other pathogens? (Section 5.2.3) Does this process of subversion place the cell at risk for cancer? 

#### 5.2.1. The Impact of Bias on Chronic Infection

We propose an expanded model for GHV latency (Figure 1) that incorporates extrafollicular B cells as a reservoir of latency that is relevant to long-term infection and disease. Much of the bias in the Ig repertoire supports a non-stochastic engagement of B cells that leads to receptor editing prior to GC entry. The virus certainly partakes in the GC, but the origin of plasmablasts that drive MHV68 reactivation at d16 could be extrafollicular or GC experienced. Extrafollicular derivation of memory may enable the virus to avoid GC selection.

BCR engagement and signaling are well-known stimuli of GHV reactivation. The observation of MHV68 accumulation in B cells that are not virus reactive would seem beneficial to the virus. This would enable the virus to avoid virus-Ag driven engagement of the BCR. Taken together, a low-level of virus-reactivity would be beneficial by promoting longevity of the latent pool. However, the infection of B cells carrying non-specific antibody would seemingly place the GHV at higher risk of elimination in the competitive GC environment. EBV is believed to rescue B cells with non-productive BCRs via surrogate signaling from LMP1 and LMP2A [225]. This might also apply to non-reactive B cells for EBV. It is not clear how the rhadinovirues would accomplish this, but they do encode a v-bcl2 that might function in this capacity [35,185].

In the GHV field, it is not clear how the proliferating population (e.g., EBV lymphocytes expanded in IM during the establishment of latency) contributes to the long-term latency in the periphery of healthy individuals later in life. In many MHV68 pathogenesis studies, there is an apparent disconnect in the phenotypes at early and late-stage infection. For instance, in the absence of a particular host gene or viral factor, a substantial defect in the establishment of splenic latency at 14–18 dpi is often observed (some examples include [226,227,228,229]). More recently, latency in particular subsets has been analyzed for such phenotypes, and the GC was found to harbor less virus [172,177]. Surprisingly, and more often than not, these large phenotypes at 16 dpi are ‘lost’ by six weeks after infection. The mutant virus or host conditions no longer differ from WT levels of latency. One interpretation of the data is that the loss of the host or viral factor leads to a specific defect in the GC compartment that does not preclude long-term latency. However, another interpretation is that an alternate GC-independent route or cell type is the source of a long-term reservoir. This alternate route is only revealed in the absence of the GC-dependent latency pool, a pool that is typically dominant in unperturbed wild-type infections. To test for this GC-independent route, the Ig repertoire should be analyzed in the infected B cells of the mutant condition at early and late times after infection. In the context of a latency defect, the remnant 16 dpi population might prove stable if those cells are resident to the lymphoid tissue or do not re-enter the GC for homeostatic maintenance. Alternatively, the virus might directly infect a memory B cell, or it might partake in an extrafollicular differentiation event. MZ B cells are well known for their ability to produce IgM+ B cells and rapidly differentiate into plasmablasts. Indeed, at 16 dpi, this pool might be a prime source for the reactivation that is observed for MHV68. Another consideration is that the source of long-term latency involves immature B cells. Immature and transitional B cells are infected by MHV68 and ablation of transitional B cells influences latency in the GC at late times after infection [35].

In the context of the host, the liaison between the virus and B cells takes on special meaning in the GC. This is a compartment of cyclical rounds of rapid proliferation, mutation, selection and apoptosis until the surface Ig has evolved into a high-affinity BCR and obtains permission to leave. Why risk crossing this dangerous terrain? Memory B cells are not only long lived, but they likely have distinct properties from naïve cells that are beneficial to the virus such as the capacity for migration, homing and self-renewal [230]. Whether extrafollicular or GC in origin, the unique functions of memory B cells that contribute to GHV pathogenesis and cancer remain ill-defined.

#### 5.2.2. BCR and Autoimmunity and Other Distractions

Viruses are factors that may trigger or exacerbate diseases that involve autoantibodies. Associations have been made between EBV and multiple sclerosis (MS) (reviewed by Ascherio and Munger [231]) and SLE [232]. Tolerance mechanisms remove self-reactive antibodies at various stages during bone marrow or GC development via central or peripheral tolerance which induces apoptosis, anergy, or receptor editing. Various defects in stages of central and peripheral tolerance are found in SLE patients [233]. How EBV plays a role is unclear, since EBV is prevalent worldwide, but associated autoimmune conditions are rare. Mouse infection models such as MHV68 demonstrate polyclonal B cell activation and a rapid increase in IgG levels, and include B cells expressing immunoglobulins to self-antigens like DNA, chromatin, collagen II [175,177,234,235,236]. However, this polyclonal B cell activation is temporary and does not persist in long-lived plasma cells [236]. 

The impact of GHV infection on autoimmune disorders is not well characterized. In studies of autoimmune-like mouse models, latent infection with MHV68 suppresses development of lupus-like disease [234] and diabetes type 1 in non-obese diabetic mice (NOD) [237]. Simultaneously, MHV68 worsens the symptoms of autoimmune encephalomyelitis (EAE) [238] and triggers relapses of autoimmune arthritis [239]. The studies by Zelazowska et al. [88] and Collins et al. [89] demonstrated that MHV68 subverts the selection process giving rise to a biased BCR repertoire. The mouse Ighv10-1 gene segment that MVH68 preferentially selects for in the GC has intrinsic autoreactive properties. The germline Ighv10-1 CDR1 and CDR2 loops have affinity for dsDNA independent of CDR3 or the light chain [219]. This brings up the intriguing possibility that MHV68 subversion of selection in the GC may disrupt tolerance and lead to the survival of B cells with autoreactive BCRs. If so, the receptor editing and lambda usage that is observed may be critical to mitigating the autoreactivity. Analysis of repertoire at early infection time points prior to receptor editing would provide further insight. 

A parallel example of murine Ighv10-1 in humans is IGHV4-34, a well-studied VH gene segment with autoreactive properties that has affinity to conserved epitopes on the surface of red blood cells, dsDNA and cardiolipin [240]. In healthy individuals, IGHV4-34 is rare due to elimination by tolerance but is high in sera from IM and SLE patients, and correlates with disease severity [241]. How and whether virus subversion of tolerance occurs is unclear in human patients. However, tracking MHV68 infection in autoimmune mice models and correlating self-reactive repertoire bias by the virus could give mechanistic insight into how viral subversion of B cell selection impacts tolerance and autoimmunity. 

#### 5.2.3. Cancer

EBV and KSHV belong to a group of seven oncoviruses that infect humans. The incidence of infection with oncoviruses far exceeds the rate of their respective cancers. Thus, the oncoviruses are likely not sufficient for, or efficient in, driving transformation. This end goal is typically not beneficial to the virus. Genetic insults driven by inherited alleles, chronic inflammation, dysregulation of immune surveillance, or environmental mutagens are co-factors in oncogenesis. GHVs may set the stage or provide the final push to transformation [242,243]. 

In the context of newly infected B cells, GHVs drive proliferation and upregulate factors key to recombination and mutation, RAG1/2 recombination factors by KSHV and AID by EBV and KSHV. The GC is a danger zone where aberrant somatic hypermutation may induce point mutations and translocations that dysregulate protooncogenes [244]. Upregulation of myc that is a hallmark of BL is caused by reciprocal chromosomal translocation between myc and Ig genes (Ig/myc) that dysregulates cell cycle and leads to tumorigenesis (reviewed in Allday [245]). Other genetic alterations include mutations in cell cycle and tumor suppressor proteins.

EBV infection can subvert the physiological selection process in the GC. During affinity maturation, the expression of functional BCR on the surface of the B cell is pivotal. Lack of sufficient signal from BCR induces negative selection via apoptosis. EBV is able to rescue Ig-deficient GC cells generated in tonsillar cultures [225,246]. Similar observations were made in patients with EBV-associated PTLD [247].

The distinct features of the two types of B cell lymphoproliferations etiologically linked to KSHV suggest an independent extrafollicular marginal zone origin of MCD and a post-germinal center origin for PEL. Detailed analysis of the Ig locus of these cells might better indicate the state of B cell differentiation that KSHV encountered. The occurrence of a dual infection of most PEL with KSHV and EBV is a fascinating scenario. EBV seems to enhance KSHV infection in cell culture and animal models, suggesting that KSHV might infect post-GC B cells already infected with EBV. In PLWH, PEL may arise concurrent to, or following, a diagnosis with MCD [107,248,249]. One study of a single patient reports no clonal relationship between MCD and PEL [107]. However, Ig repertoire analysis of a larger cohort would more definitely address a potential clonal linkage between MCD and PEL that would clarify the origin of PEL.

Single-cell sequencing provides the resolution needed to dissect the intricacies of GHV–host interactions. Isolation of individual infected B cells will allow us to identify B cell compartments susceptible to viral infection as well as viral expression programs governing the fate of the target cell. Simultaneous sequencing of the genome and transcriptome of the host and pathogen will also be critical for understanding virus latency, autoimmunity, and viral oncogenesis. Recently, these methods were used with success to study memory B cells generated in response to influenza virus [250] as well as the transcription profile of Dengue virus [251]. Single-cell genomics and transcriptomics is revealing novel functions of tumor infiltrating immune cells [252], recently in EBV-associated carcinoma [253]. In parallel, new techniques are being developed to allow high-throughput sorting and pairing of Ig heavy and light chain from isolated B cells [254]. The field is on the verge of an exciting explosion of integrated viral and host genomics datasets.

## Figures and Tables

**Table 1:** B cell repertoire of non-transformed GHV-infected cells.

Virus	Source	Tissue	#Cells	Bias	Ref.
**EBV**	IM patients	PB (CD20+, CD27+)	32	V_H_ mutated evidence of antigen selection	[80]
	IM patients	PB (CD20+, CD27+)	100–300	more SHM events in EBV+ EBV excluded from IgM+	[81]
	IM patients	CD19+, IgD−,	56 Abs ^a^	no bias in IGHV usage, V_H_ mutated evidence of antigen selection	[82]
	IM patients	sera		elevated level of IGHV4-34 expressing Abs ^b^	[83]
	IM patients	sera		elevated level of IGHV4-34 expressing Abs ^b^	[84]
	in vitro infection	healthy PBMCs, naïve (IgD+, CD27−)	25 ^c^	accumulation of SHM events with time, no CSR detected, clonal expansion	[79]
	in vitro infection	healthy PBMCs, non-switched memory (IgD+, CD27+)	38 ^c^	SHM pattern does not change with time, no CSR detected	[79]
	in vitro infection	healthy PBMCs, switched memory (IgD−, CD27+)	55 ^c^	SHM pattern does not change with time, no CSR detected	[79]
	in vitro infection	healthy PBMCs		bias in IGHV usage, lower BCR diversity, dominant consensus CDR3 motif	[85]
**KSHV**	in vitro infection	tonsillar B cells		infection restricted to IgMλ B cells	[86]
	in vitro infection	naïve B cells from tonsils (CD38^low^IgD+CD27−)	480	induction of Igλ expression, bias in IGLV4 usage	[87]
**MHV68**	C57Bl/6	MHV+ GC cells (CD19+GL7^high^CD95+)	>400 ^d^	bias Ighv10 usage, bias in Igλ usage	[88]
	C57Bl/6	MHV+ GC cells (CDB220+GL7^high^CD95+) MHV+ PC (B220^lo-neg^, CD138+)	100–200 ^d^	bias Ighv10 usage, bias in Igλ usage	[89]

^a^ Number of antibodies cloned from EBV+ memory B cells; ^b^ measured by reactivity with 9G4 antibody (anti-IGHV4-34); ^c^ number of cultures analyzed; ^d^ number of V_H_ sequences analyzed; IM—infectious mononucleosis, PBMCs—peripheral blood mononuclear cells, GC—germinal center, PC—plasma cells, and SHM—somatic hypermutation.

**Table 2:** BCR repertoire of gammaherpesvirus-associated cancers.

Virus	Cancer	Source	#Patients	Remarks	Ref.
**EBV**	eBL	EBV+ tumor samples	11 tumor samples	IGHV1-69, IGHV3-23 and IGHV4-34 overrepresented in eBL	[202]
	BL	EBV+ tumor samples	71 tumor samples	IGHV4-34 and IGHV3-30 overrepresented	[199]
	NPC	sera		elevated level of IGHV4-34 expressing Abs ^a^	[84]
	CLL	PBMCs	25 patients positive for either EBV or CMV	higher frequency of IGHV4-34 ^b^	[192]
	CLL	PBMCs	9 patients positive for both EBV and CMV	exclusive expression of IGHV4-34 ^b^	[192]
	PTLD (P-PTLD, DLBCL, BL)	tissue sections	26 patients	no IGHV bias detected, crippled BCR present	[206]
**KSHV**	KSHV+ MCD	tissue sections	13 patients	no bias in IGHV; λ light chain restriction; low SHM	[22]
	PEL	cell samples of lymphomatous effusions	4 patients	monoclonal Ig expressing IGHV3-23, IGHV3-73, IGHV4-39, and IGHV1-03, bias in λ usage	[24]
	PEL cell lines	cell line	3 cell lines	monoclonal Ig expressing IGHV3-73 and IGHV5-51	[24]

^a^ Measured by reactivity with 9G4 antibody (anti-IGHV4-34); ^b^ detected by PCR; eBL—endemic Burkitt’s lymphoma, BL—Burkitt’s lymphoma, NPC—nasopharyngeal carcinoma, CLL—chronic lymphocytic leukemia, PBMCs—peripheral blood mononuclear cells, CMV—cytomegalovirus, PTLD—post-transplant lymphoproliferative disorder, P-PTLD—polymorphic post-transplant lymphoproliferative disorder, DLBCL—diffuse large B cell lymphoma, MCD—multicentric Castleman disease, and PEL—primary effusion lymphoma.
